# Addressing the Relationship between Leaf Nitrogen and Carbon Isotope Discrimination from the Three Levels of Community, Population and Individual

**DOI:** 10.3390/plants12071551

**Published:** 2023-04-04

**Authors:** Shuhan Wang, Yaowen Han, Yufu Jia, Zixun Chen, Guoan Wang

**Affiliations:** 1 Beijing Key Laboratory of Farmland Soil Pollution Prevention and Remediation, College of Resources and Environmental Sciences, China Agricultural University, Beijing 100193, China; 2 Department of Biotechonology, College of Biotechonology and Pharmceutical Engineering, Nanjing Tech University, Nanjing 211816, China; 3 Institute of Botany, The Chinese Academy of Sciences, Beijing 100093, China

**Keywords:** leaf nitrogen, carbon isotope discrimination, relationship, community, population, individual

## Abstract

The carbon, nitrogen and water cycles of terrestrial ecosystems are important biogeochemical cycles. Addressing the relationship of leaf nitrogen (N) and carbon isotope discrimination (Δ) will enhance the understanding of the links between these three cycles in plant leaves because Δ can reflect time-integrated leaf-level water-use efficiency (WUE) over the period when the leaf material is produced. Previous studies have paid considerable attention to the relationship. However, these studies have not effectively eliminated the interference of environmental factors, inter-species, and inter-individual differences in this relationship, so new research is necessary. To minimize these interferences, the present work explored the relationship at the three levels of community, population, and plant individual. Three patterns of positive, negative and no relationship were observed across communities, populations, and individuals, which is dependent on environmental conditions, species, and plant individuals. The results strongly suggested that there is no general pattern for the relationship between leaf N and Δ. Furthermore, the results indicated that there is often no coupling between leaf-level long-term WUE and leaf N in the metabolic process of carbon, N and water in leaves. The main reason for the lack of this relationship is that most plants do not invest large amounts of nitrogen into photosynthesis. In addition, the present study also observed that, for most plant species, leaf N was not related to photosynthetic rate, and that variations in photosynthetic rates are mainly driven by stomatal conductance.

## 1. Introduction

Carbon-isotope discrimination (Δ) in plants is associated with photosynthetic rate (A) and stomatal conductance (g_s_) [1,2,3,4]; photosynthetic rate varies with leaf N concentrations because most leaf N concentrates in the photosynthetic apparatus [5,6,7]; hence, a tight relationship between leaf N and Δ is expected. Numerous studies have confirmed this relationship [8,9,10,11,12,13]. However, lack of this relationship has also been observed in a few investigations [14,15]. The inconsistent results indicate that there is still a lot of uncertainty with respect to the relationship between leaf N and Δ.

Leaf N and Δ both are affected by many environmental factors and are species-specific and individual-specific dependent [8,12,16,17,18,19,20,21], so changes in environmental factors, plant species and individual may have a great impact on the relationship between leaf N and Δ. Spatial investigations along an environmental gradient, such as investigations along an altitude gradient, often involve changes in multiple environmental factors; furthermore, vegetation types and species also change with environmental gradients. Thus, this commonly used investigation along environmental gradients cannot effectively eliminate the interferences of environmental factors, inter-species, and inter-individual differences with the relationship between leaf N and Δ, resulting in uncertainty in the relationships between leaf N and Δ. New studies that minimize these interferences will therefore better constrain this relationship.

Carbon-isotope discrimination in plants is a good proxy for time-integrated leaf-level water-use efficiency (WUE) over the period when the leaf material is produced [1,12,22,23,24,25,26]. WUE is defined as the amount of organic carbon produced by consuming per unit water, which reflects carbon–water balance and cycles of carbon and water of terrestrial ecosystems. Leaf N is associated with the absorption and allocation of N by plants, which are key processes of the N cycle of terrestrial ecosystems. Thus, constraint of this relationship between leaf Δ and N could contribute to a better understanding of the connection between the carbon, nitrogen and water biogeochemical cycles of terrestrial ecosystems [27]. 

As stated above, there should be a closed link between leaf N and Δ due to the key role of leaf N in the synthesis of the photosynthetic apparatus [5,6,7]. However, the interference of environmental factors, inter-species and inter-individual differences obscure the relationship between leaf N and Δ, resulting in the irrelevance observed by a previous study [14,15]. Therefore, we hypothesized that Δ is positively correlated with leaf N when the effect of environmental factors, inter-species and inter-individual differences upon this relationship has been minimized. To test our hypothesis, the present study explored the relationship from the three levels of community, population, and plant individual. Our aim was to effectively constrain the relationship between leaf N and Δ and raise awareness of the biogeochemical cycles of carbon, nitrogen, and water in terrestrial ecosystems.

## 2. Results

**Figure 1:**
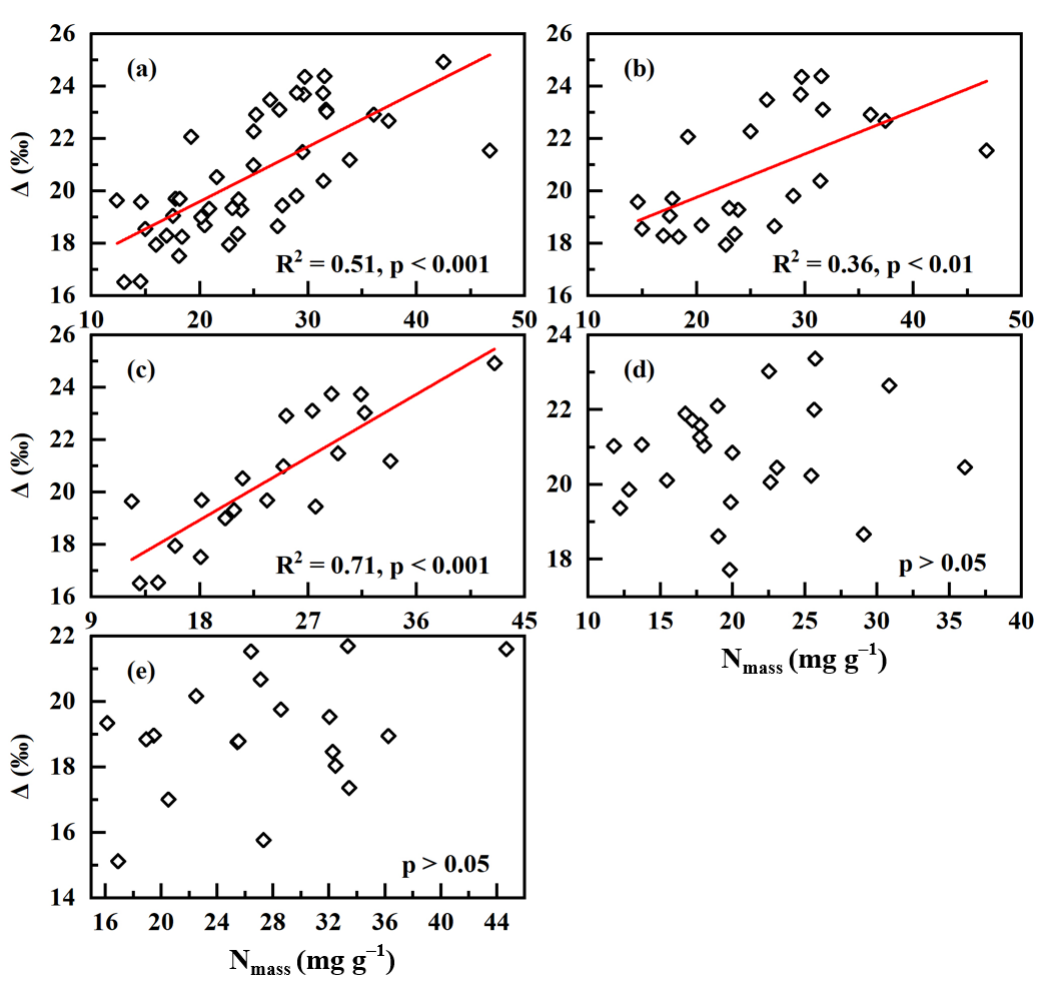
Relationship between leaf N based on mass (N_mass_) and carbon-isotopic discrimination (Δ) at the community level for plants derived from the first sampling campaign: (**a**) Baiwangshan mountain; (**b**) the sunny slope of Baiwangshan mountain; (**c**) the shaded slope of Baiwangshan mountain; and (**d**) Luochuan, (**e**) Shapotou.

At the community level, the forestry vegetation on Baiwang Mountain showed a significant positive relationship between N based on leaf mass (N_mass_) and Δ (R^2^ = 0.51, *p* < 0.001; Figure 1a); in detail, the vegetation on the shaded slope yielded a more strong positive relationship than that on the sunny slope (Figure 1b,c; R^2^ = 0.36, *p* < 0.01 for the sunny slope and R^2^ = 0.71, *p* < 0.001 for the shaded slope). Leaf N_mass_ was not related to Δ for both the herbaceous vegetation in Luochuan and the grass-desert vegetation in Shapotou (*p* > 0.05 for both; Figure 1d,e).

**Figure 2:**
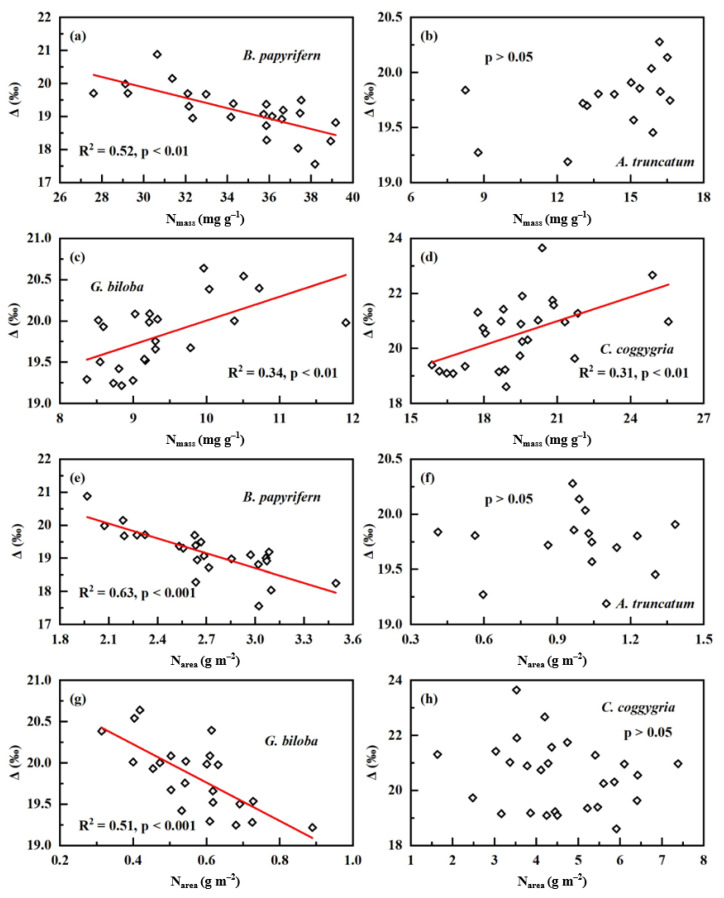
Relationships between leaf N and carbon isotopic discrimination (Δ) at the population level for plants derived from the second sampling campaign: (**a**–**d**) N_mass_ vs. Δ; and (**e**–**h**) N_area_ vs. Δ.

At the population level, Δ was negatively correlated with both N_mass_ and N based on leaf area (N_area_) for *Broussonetia papyrifera* (*B. papyrifern*, R^2^ = 0.52, *p* < 0.01 for N_mass_ vs. Δ; R^2^ = 0.63, *p* < 0.001 for N_area_ vs. Δ; Figure 2a,e). *Acer truncatum* (*A. truncatum*) did not show any relationship between Δ and the two N parameters (*p* > 0.05 for both; Figure 2b,f). Δ was positively related to N_mass_ and negatively to N_area_ for *Ginkgo biloba* (*G. biloba*, R^2^ = 0.34, *p* < 0.01 for N_mass_ vs. Δ; R^2^ = 0.51, *p* < 0.001 for N_area_ vs. Δ; Figure 2c,g). *Cotinus coggygria* (*C. coggygria*) had a positive relationship between N_mass_ and Δ (R^2^ = 0.31, *p* < 0.01; Figure 2d), but lacked the relationship between N_area_ and Δ (*p* > 0.05; Figure 2h).

**Figure 3:**
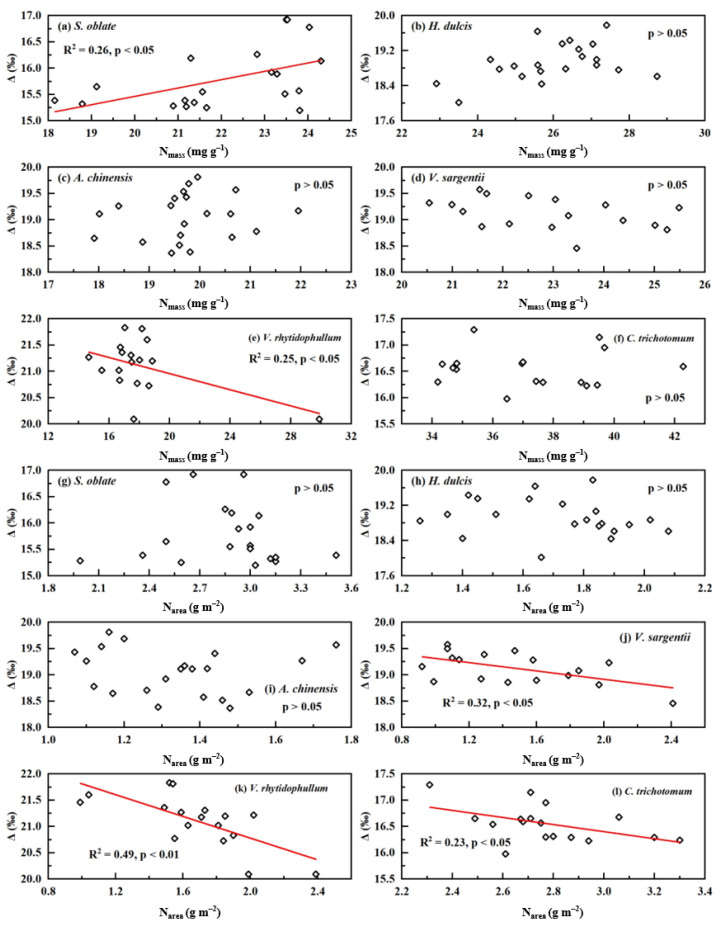
Relationships between leaf N and carbon isotopic discrimination (Δ) at plant individual level for plants derived from the third sampling campaign: (**a**–**f**) N_mass_ vs. Δ; and (**g**–**l**) N_area_ vs. Δ.

At the plant individual level, only *Syringa oblate* (*S. oblate*) showed a positive correlation between N_mass_ and Δ (R^2^ = 0.26, *p* < 0.05, Figure 3a), and only *Viburnum rhytidophyllum* (*V. rhytidophyllum*) showed a negative correlation between N_mass_ and Δ (R^2^ = 0.25, *p* < 0.05, Figure 3e), while with the other four species, N_mass_ was not related to Δ (*p* > 0.05 for all; Figure 3b–d,f). For *Viburnum sargentii* (*V. sargentii*), *V. rhytidophyllum* and *Clerodendrum trichotomum* (*C. trichotomum*), there were significantly negative correlations between N_area_ and Δ (R^2^ = 0.32, *p* < 0.05 for *V. sargentii*, R^2^ = 0.49, *p* < 0.01 for *V. rhytidophyllum*, and R^2^ = 0.23, *p* < 0.05 for *C. trichotomum*, Figure 3j–l), while none of the other three species had that correlation (*p* > 0.05 for all species, Figure 3g–i).

**Figure A1:**
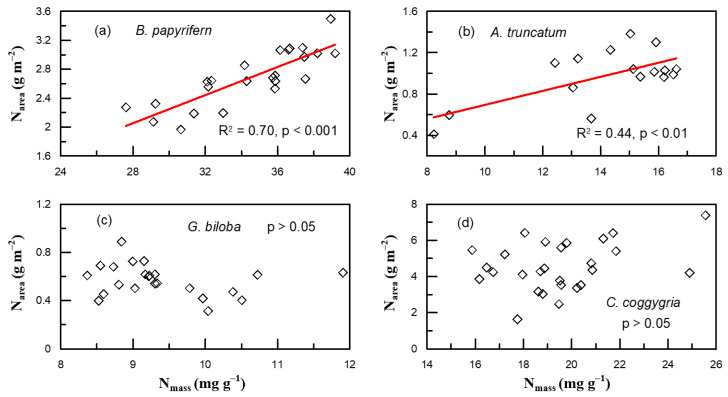
Relationship between leaf N based on area (N_area_) and leaf N based on mass (N_mass_) of four species derived from the second sampling campaign: (**a**) *B. papyrifern*, (**b**) *A. truncatum*, (**c**) *G. biloba*, (**d**) *G. coggygria*.

**Figure A2:**
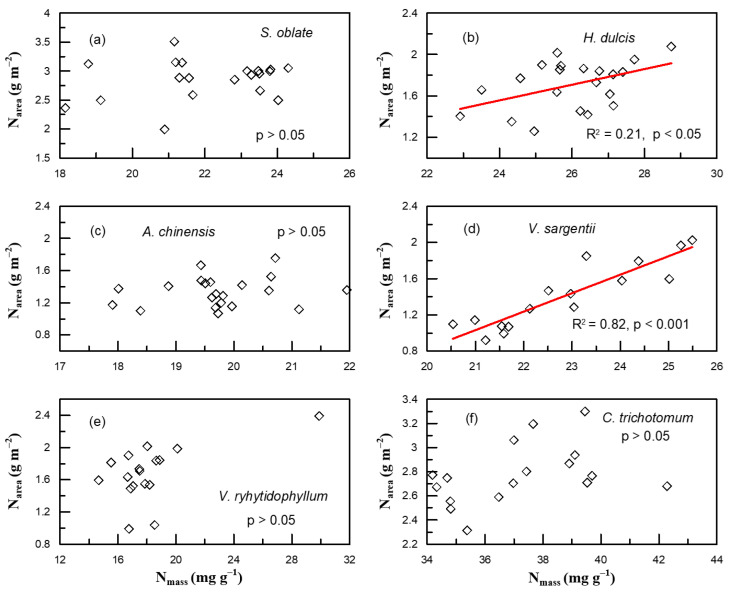
Relationship between leaf N based on area (N_area_) and leaf N based on mass (N_mass_) of six species derived from the third sampling campaign: (**a**) *S. oblate*, (**b**) *H. dulcis*, (**c**) *A. chinensis*, (**d**) *V. sargentii*, (**e**) *V. rhytidophyllum*, (**f**) *C. trichotomum*.

Although N_area_ and N_mass_ both express leaf N concentration and can be interconverted via the leaf mass per unit area, at population level, only *B. papyrifern* and *A. truncatum* displayed a positive correlation between N_area_ and N_mass_ (R^2^ = 0.70, *p* < 0.001 for *B. papyrifern*; R^2^ = 0.44, *p* < 0.01 for *A. truncatum*; Figure A1a,b); at the plant individual level, only *Hovenia dulcis* (*H. dulcis*) and *V. sargentii* showed a positive relationship (R^2^ = 0.21, *p* < 0.05 for *H. dulcis*; R^2^ = 0.82, *p* < 0.001 for *V. sargentii*; Figure A2b,d); the others all lacked the relationship (*p* > 0.05 for all species, Figure A1 and Figure A2).

**Figure 4:**
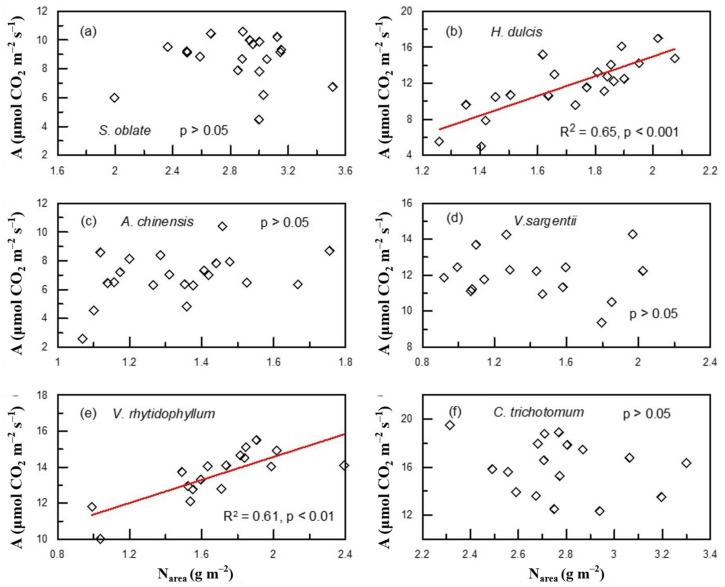
Relationships between leaf N based on area (N_area_) and photosynthetic rate (A) on plant individual level for plants derived from the third sampling campaign: (**a**) *S. oblate*, (**b**) *H. dulcis*, (**c**) *A. chinensis*, (**d**) *V. sargentii*, (**e**) *V. rhytidophyllum*, (**f**) *C. trichotomum*.

**Figure 5:**
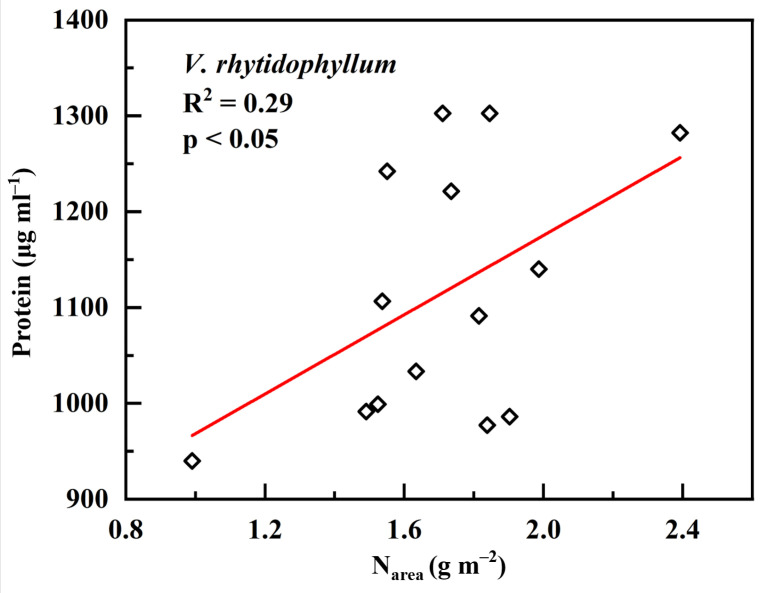
Relationship between leaf N based on area (N_area_) and protein concentration in *V. rhytidophyllum*.

**Figure 6:**
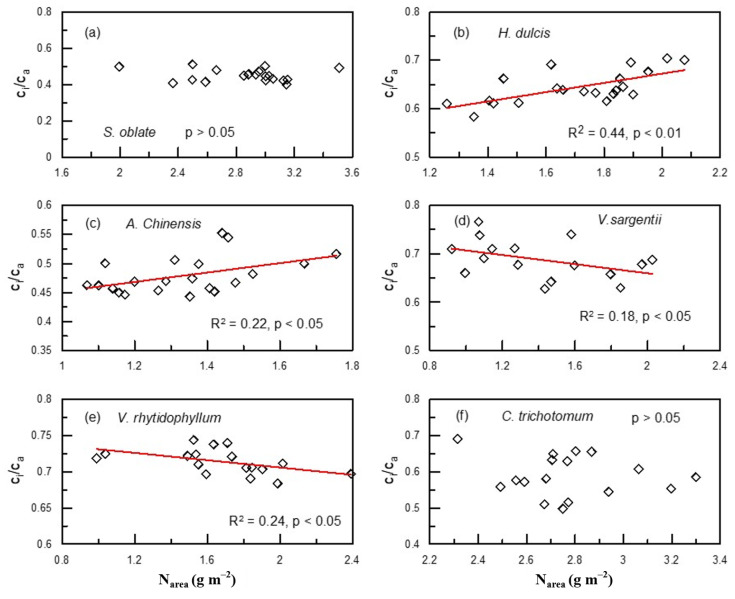
Relationships between leaf N based on area (N_area_) and the ratio of intercellular to ambient CO_2_ concentration (c_i_/c_a_) at plant individual level for plants derived from the third sampling campaign: (**a**) *S. oblate*, (**b**) *H. dulcis*, (**c**) *A. chinensis*, (**d**) *V. sargentii*, (**e**) *V. rhytidophyllum*, (**f**) *C. trichotomum*.

**Figure 7:**
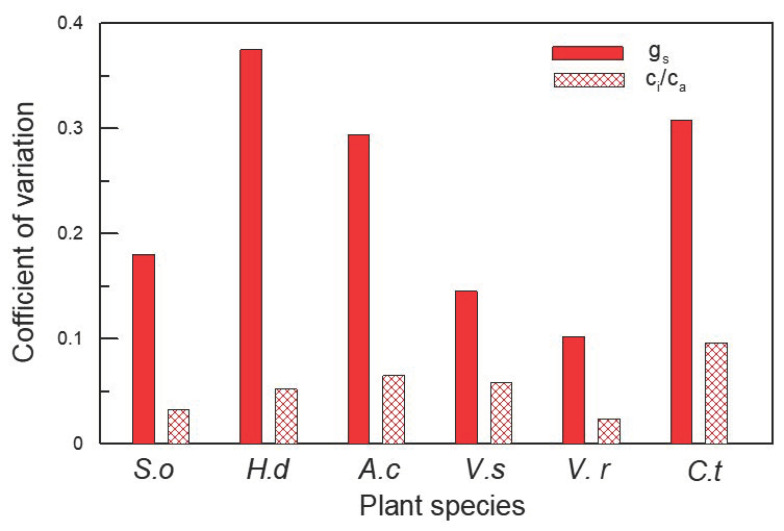
Coefficient of variation of stomatal conductance (g_s_) and the ratio of intercellular to ambient CO_2_ concentration (c_i_/c_a_) for plants derived from the third sampling campaign. Abbreviation: *S.o*, *S. oblate*; *H.d*, *H. dulcis*; *A.c*, *A. chinensis*; *V.s*, *V. sargentii*; *V.r*, *V. rhytidophyllum* and *C.t*, *C. trichotomum*.

**Figure 8:**
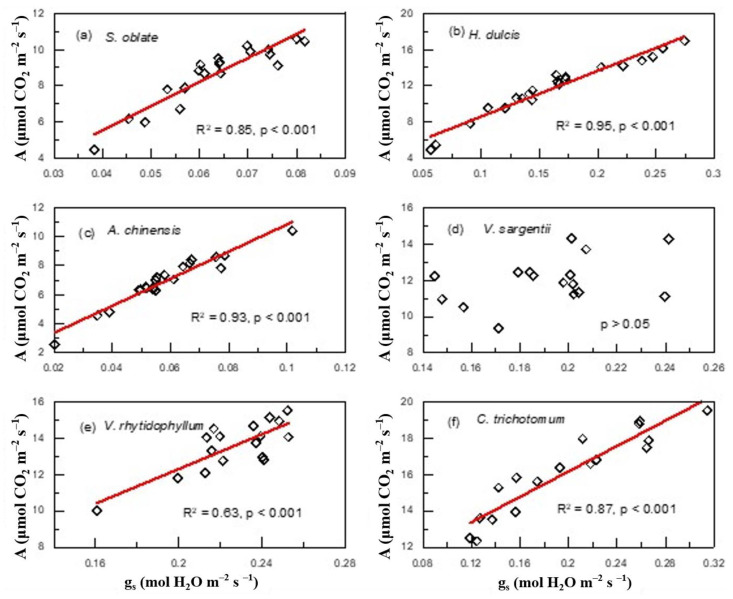
Relationships between stomatal conductance (g_s_) and photosynthetic rate (A) at plant individual level for plants derived from the third sampling campaign: (**a**) *S. oblate*, (**b**) *H. dulcis*, (**c**) *A. chinensis*, (**d**) *V. sargentii*, (**e**) *V. rhytidophyllum*, (**f**) *C. trichotomum*.

Of the six plant species that have undergone gas-exchange measurements, only *H. dulcis* and *V. rhytidophyllum* had a positive correlation between N_area_ and photosynthetic rate (A) (Figure 4b,e; R^2^ = 0.65, *p* < 0.001 for *H. dulcis*; R^2^ = 0.61, *p* < 0.001 for *V. rhytidophyllum*). The other four species did not show the relationship (Figure 4a,c,d,f; *p* > 0.05 for all). Only *V. rhytidophyllum* had a positive correlation between N_area_ and protein concentration (Figure 5, R^2^ = 0.29, *p* < 0.05). The other five plants showed no correlation (Table A1; *p* > 0.05 for all). There were three patterns of the relationship between N_area_ and the ratio of intercellular to ambient CO_2_ concentration (c_i_/c_a_), positive relationship for *H. dulcis* (Figure 6b; R^2^ = 0.44, *p* < 0.01) and *Aesculus chinensis* (*A. chinensis*, Figure 6c; R^2^ = 0.22, *p* < 0.05), negative relationship for *V. sargentii* (Figure 6d; R^2^ = 0.18, *p* < 0.05) and *V. rhytidophyllum* (Figure 6e; R^2^ = 0.22, *p* < 0.05), and no relationship for *S. oblate* and *C. trichotomum* (Figure 6a, f; *p* > 0.05 for both). All six species displayed a small coefficient of variation in c_i_/c_a_ (Figure 7). They were 0.033, 0.052, 0.065, 0.058, 0.024 and 0.096 for *S. oblate*, *H. dulcis*, *A. chinensis*, *V. sargentii*, *V. rhytidophyllum* and *C. trichotomum*, respectively. Compared to c_i_/c_a_, stomatal conductance (g_s_) varied much more drastically (Figure 7). The coefficient of variation was 0.18, 0.38, 0.29, 0.15, 0.10 and 0.31 for *S. oblate*, *H. dulcis*, *A. chinensis*, *V. sargentii*, *V. rhytidophyllum* and *C. trichotomum*, respectively. *V. sargentii* lacked a correlation between A and g_s_ (Figure 8d, *p* > 0.05), while the other five species all showed a highly significant positive relationship (Figure 8a–c,e,f; R^2^ = 0.85, *p* < 0.001 for *S. oblate*; R^2^ = 0.95, *p* < 0.001 for *H. dulcis*; R^2^ = 0.93, *p* < 0.001 for *A. chinensis*; R^2^ = 0.54, *p* < 0.01 for *V. rhytidophyllum*; R^2^ = 0.73, *p* < 0.001 for *C. trichotomum*).

**Figure A3:**
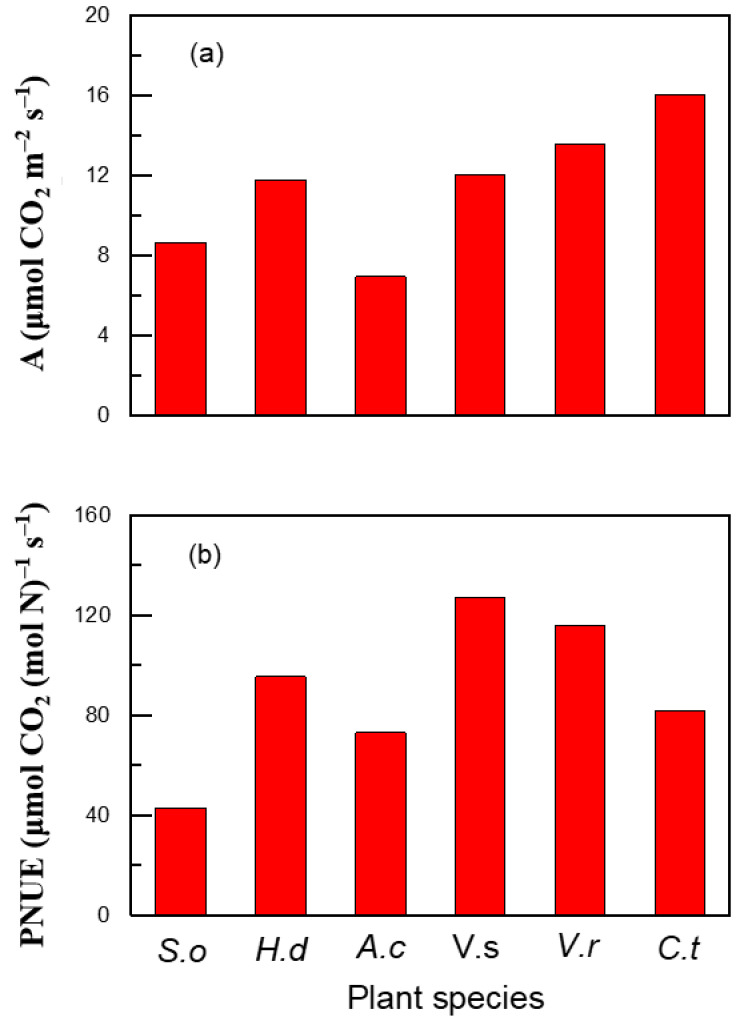
The average photosynthetic rate (A) (**a**) and photosynthetic N use efficiency (PNUE) (**b**) of six species derived from the third sampling campaign. Abbreviation: *S. oblate (S.o), H. dulcis (H.d), A. chinensis (A.c), V. sargentii (V.s), V. rhytidophyllum (V.r)* and *C. trichotomum (C.t)*.

We also assessed the photosynthetic N use efficiency (PNUE) of six plant species grown in the campus of CAU. PNUE is defined as the ratio of A and leaf N_area_, and it also links carbon cycle and nitrogen cycle in plants. The highest PNUE (126.95 μmol CO_2_ (mol N)^−1^ s^−1^) was in *V. sargentii*; the second highest was in *V. rhytidophyllum* (115.78 μmol CO_2_ (mol N)^−1^ s^−1^). The PNUE was 42.86 μmol CO_2_ (mol N)^−1^ s^−1^, 95.36 μmol CO_2_ (mol N)^−1^ s^−1^, 72.86 μmol CO_2_ (mol N)^−1^ s^−1^ and 81.78 μmol CO_2_ (mol N)^−1^ s^−1^ for *S. oblate*, *H. dulcis*, *A. chinensis* and *C. trichotomum*, respectively (Figure A3).

## 3. Discussion

This study explored the relationship between leaf N and Δ from the three levels of community, population, and individual. At the community level, we observed two patterns of positive correlation and no correlation across three different sites (Figure 1). Within each study site, the environmental factors were similar for all plants; thus, the observed relationship between leaf N and Δ based on the community scale in the same site was environmentally independent. This relationship varies across different study sites, indicating that the close correlation between leaf N and Δ or WUE is not a general pattern at the community scale when minimizing the interference of environmental conditions. For a given community, whether Δ are leaf N-dependent may be related to its plant composition and local environmental conditions. At the population level, three patterns, positive, negative and no relationship between leaf N and Δ, were observed for four plant species grown in the same environmental conditions (Figure 2). This investigation, based on the population level, ruled out the effect of environmental factors and the interference of inter-species difference on the relationship, and thus our observation suggests that there is no consistent leaf N–Δ correlation at the population level. It should be pointed out that the leaf samples presented at the population level were collected in November, and were in the senescence phase of plants in the northern hemisphere. Previous studies have shown that leaf-nutrient contents change across growth stages and are lowest in the senescence stage [28,29]. However, decreasing N contents in the senescence phase occurred in all samples presented at the population level; thus, leaf N–Δ relationship may not be affected by the decreases in N contents. At the individual level, this study also yielded three different patterns for six species grown at the campus of CAU (Figure 3). For each plant species selected, we collected leaf samples from only one individual, therefore this investigation illustrated that the leaf N–Δ relationship at the individual level is also not consistent when excluding the interference of environmental factors, inter-species difference and inter-individual differences. As mentioned above, environmental factors, species difference and individual differences affect leaf N and Δ, and thus the relationship between them. Most previous studies have not effectively eliminated these interferences; therefore, this study better reveals the relationship between leaf N and Δ compared with previous results. Our results contradict our previous hypothesis, and strongly suggest that there is not a general pattern for the relationship between leaf N and Δ, which is dependent of environmental conditions, species, and plant individuals. Since leaf Δ reflects long-term leaf-level water-use efficiency (WUE) [1,22], our observation indicates that long-term leaf-level WUE may be often leaf N-independent in the metabolic process of carbon, N and water in leaves.

Previous studies [5,30,31,32] suggested that the photosynthetic apparatus contain most leaf N, and thus A increases and c_i_/c_a_ decreases with increasing leaf N. However, in the present study, only *H. dulcis* and *V. rhytidophyllum* had a positive correlation between N and A (Figure 4), and only *V. sargentii* and *V. rhytidophyllum* showed a negative correlation between N and c_i_/c_a_ (Figure 6). The finding indicates that for most plants, the photosynthetic apparatus is not the largest N sink in plants. Most enzymes involved in photosynthesis are proteins, so Figure 5 and Table A1 suggested that except *V. rhytidophyllum*, the leaf N is not mainly used to synthesize photosynthetic enzymes for the other five plants. This result further confirms the conclusion that most N is not allocated into the photosynthetic apparatus for most plants. Large amounts of nitrogen in plants could be used to synthesize nitrogenous secondary compounds, such as alkaloid and cyanogenic glycoside, or be stored in nitrate and organic N, all of which are not associated with photosynthetic capacity. In addition, leaf N may invest in seed and herbivore defense [11]. Furthermore, N could also be invested in roots through amino acid biosynthesis, especially in plant-rhizobium and plant-mycorrhiza symbiotic systems [33,34]. Given that there is always a tight correlation between Δ and A and c_i_/c_a_, the fact that leaf N does not concentrate in the photosynthetic apparatus led to the lack of a relationship between leaf N and Δ in these plants. 

In addition to being regulated by leaf N allocation, the relationship between leaf N and Δ may also be controlled by mesophyll conductance (g_m_). High internal resistance (i.e., low g_m_) can increase the effect of internal resistance on CO_2_ concentration (c_c_) at the sites of carboxylation and reduce the control of the photosynthesis rate on c_c_, which may eventually cause leaf N unrelated to Δ [35]. Many plant species were reported to have small g_m_ [36,37,38,39,40,41,42]. Thus, we speculate that plants lacking the relationship between N and Δ could have small g_m_. Unfortunately, we did not perform the g_m_ measurements in this study. Our future work will add this measurement.

Since A was not related to leaf N for most of the plants we investigated, it raises a question as to what drives variations in A. This study demonstrated that changes in A were mainly driven by changes in g_s_, because the c_i_/c_a_ ratio showed much smaller changes compared to g_s_ (Figure 7); moreover, except for *V. sargentii*, the other plants all had an extremely significant positive relationship between A and g_s_ (Figure 8). The strong correlation between A and g_s_ was also found to hold across large number of species and life forms [43,44,45]. Furthermore, g_s_ was regulated by environmental factors [46,47,48,49], which may also contribute to the lack of the leaf N–Δ relationship.

Leaf N allocation to the photosynthetic apparatus determines the light-saturated photosynthetic rate and photosynthetic N use efficiency (PNUE) [29,50,51]. Thus, it is expected that those plants with a tight association of N and A will have high A and PNUE because most leaf N of these plants might concentrate in photosynthetic apparatus. This study confirmed this to some extent. As aforementioned, *V. rhytidophyllum* and *H. dulcis* had a tight coupling between N and A (Figure 4), they therefore showed high A and PNUE (Figure A3). Strangely, A in *C. trichotomum* and PNUE in *V. sargentii* were higher than that in *V. rhytidophyllum* and *H. dulcis* although the two plants did not have a close coupling between N and A (Figure 4). This could be because although the observed A and c_i_/c_a_ were from eight gas-exchange measurements during the experiment, they might not be exactly equal to those occurring over the entire growth period, while the observed leaf N came from measurement of the leaves harvested on the last day of the experiment, which reflected the long-term N status. As a result, there might be a certain degree of difference between the observed relationship between leaf N and A and the actual relationship; the observed PNUE might also differ from the actual value to some extent.

## 4. Materials and Methods

### 4.1. Plant Sampling Campaigns

**Figure 9:**
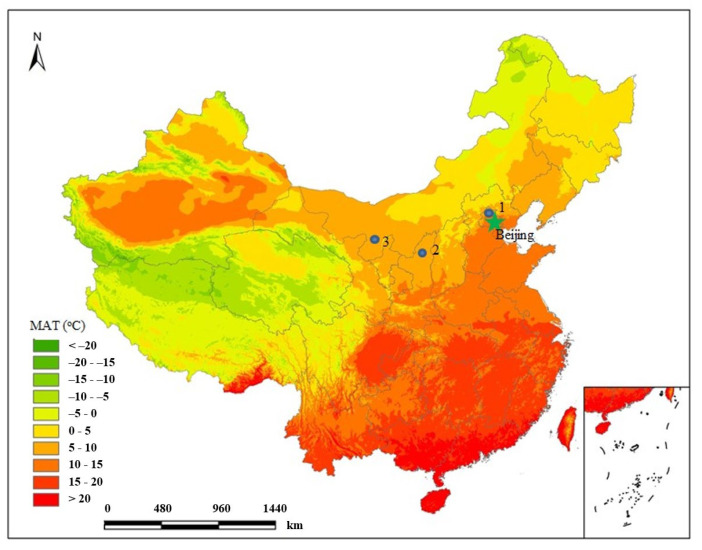
Plant sampling sites. Sampling sites are indicated with solid circles and numbers, 1, Baiwangshan Mountain, northern China; 2, Luochuan, northwestern China; 3, Shapotou, northwestern China. MAT is the mean annual temperature of the past 50 years.

We conducted three campaigns for plant sampling. The first sampling campaign was conducted in three regions with different climate conditions and vegetation types, Baiwangshan mountain, Luochuan and Shapotou. This sampling campaign aimed to explore the relationship between leaf N and Δ at the community level. The sampling at Baiwangshan mountain was performed on 15 July 2017. Baiwangshan mountain (40°01′27″ N, 116°16′21″ E) is located in the Haidian district of Beijing, northern China, about 10 km away from the center of Beijing (Figure 9). Many kinds of woody species occur in Baiwangshan mountain. The highest peak of Baiwangshan mountain is 220 m above sea level (a.s.l.) and its average altitude is about 150 m. The vegetation type is mixed deciduous forest. The common tree species are *Platycladus orientalis*, *Broussonetia papyrifera*, *Quercus aliena*, *Quercus variabilis*, *Acer truncatum*, *Koelreuteria paniculata*, *Pinus tabuliformis*, *Cotinus coggygria* and *Ginkgo biloba*, etc.; the dominant shrub species are *Vitex negundo var. heterophylla* and *Ziziphus jujuba var. spinosa*. The soil type is cinnamon-soil (luvisols). Baiwangshan mountain is characterized by a temperate semi-humid monsoon climate with a mean annual temperature (MAT) of 10 °C and a mean annual precipitation (MAP) of 600 mm. The sampling in Luochuan was conducted on 13 September 2017. The sampling site (35°42′23″ N, 109°18′20″ E, 1000 m a.s.l.) is on a small barren loess platform (~25 m × ~100 m) in Luochuan county, northwestern China, and is about 10 km away from the nearest city, Luochuan (1000 m a.s.l.) (Figure 9). The local vegetation type is temperate grassland, and a lot of herbaceous plants grow here. Luochuan is also characterized by a temperate semi-humid monsoon climate with the MAT of 9.2 °C and the MAP of 620 mm. The dominant plants were *Stipa capillata*, *Artemisia lavandulaefolia*, *Lespedeza floribunda*, *Bothriochloa ischcemum*, *Potentilla chinensis*, and *Setaria viridis*. The soil type is calcisols. The sampling at Shapotou was conducted on 15 September 2017. Shapotou (37°27′ N, 104°57′ E, 1250 m a.s.l.) is located in the Ningxia Hui Autonomous Region, northwestern China, and is about 20 km away from the nearest city, Zhongwei (Figure 9). Shapotou is characterized by a temperate dry monsoon climate with the MAT of 8.5 °C and the MAP of 186 mm. The soil type is xerosols. The sampling site is in the experimental plot (~400 m × ~1000 m) of the Shapotou Desert Research Experimental Station, Chinese Academy of Sciences, which has been fenced off since 2001 to prevent human disturbance. The site has a vegetation type of grass-desert with both shrubs and herbs. The dominant species were *Caragana korshinskii*, *Sarcozygium xanthoxylo*, *Ephedra equisetina*, *Salsola collina*, *Tamarix chinensis*, *Corispermum puberulum*, and *Reaumuria songarica*. There is a small amount of C_4_ plant species in these three sampling sites, especially in Shapotou, but the proportion of C_4_ biomass in the local community is very low, so this study focused on C_3_ plants. On Baiwangshan mountain, we set up a plot (100 m × 150 m) on the sunny slope and the shady slope respectively. All C_3_ plant species within each plot have been sampled. Depending on leaf size, 3~20 mature and healthy leaves (mostly 8) from different individuals were collected. The leaves from each species were pooled into one sample. We collected 24 and 20 samples on the sunny slope and the shady slope, respectively. The 44 plant samples covered all major local C_3_ plant species. At Luochuan, only one plot (25 m × 50 m) was set up, and 24 plant samples were collected including all major local C_3_ herbaceous species. At Shapotou, we set up one plot (200 m × 200 m) and collected 19 samples covering all major local C_3_ herbs and shrubs. The method of sampling is the same for this sampling campaign, and we collected only plant leaves. For shrubs, we collected leaves from the upper end of the branches of each individual. For tree, we sampled leaves from positions of full irradiance about 4 m above the ground. 

The second sampling campaign was conducted on 2 November 2018. The purpose of this sampling was to explore the relationship between leaf N and Δ at population level. The sampling was also carried out on Baiwangshan mountain. We set up one plot (25 m × 100 m) on a ridge. The leaves of four tree species, *Broussonetia papyrifera*, *Acer truncatum*, *Ginkgo biloba* and *Cotinus coggygria*, were collected. We chose four individuals for each species to collect samples. 24, 16, 23 and 27 healthy mature leaves were sampled from *B. papyrifera, A. truncatum, G. biloba* and *C coggygria*, respectively. A total of 4–7 leaves of each plant individual from the positions of full irradiance, about 4–5 m above the ground were collected. One leaf makes up one sample.

The third sampling campaign was performed on 18 July 2019. The aim of this sampling was to evaluate the relationship between leaf N and Δ at individual level. The sampling site is in the campus of China Agricultural University (CAU, 40 m a.s.l.), which is located in the Haidian district of Beijing (40°01′27″ N, 116°16′22″ E). Three tree species, *Syringa oblate, Hovenia dulcis* and *Aesculus chinensis,* and three shrub species, *Viburnum sargentii, Viburnum rhytidophyllum* and *Clerodendrum trichotomum*, were chosen. The leaves of these six plants are all larger, which is conducive to our gas-exchange measurements. They all grow in the same environment. Only one plant individual was selected for each species. After the final measurement of gas-exchange (i.e., 18 July 2019), we harvested 20, 21, 21, 17, 17 and 17 leaves from *S. oblate, H. dulcis, A. chinensis, V. sargentii, V. rhytidophyllum* and *C. trichotomum*, respectively, All leaves were healthy and obtained full irradiance. Each sample consisted of one leaf.

### 4.2. Measurements of Leaf Gas-Exchange

We performed gas-exchange measurements on the 6 plant individuals mentioned above, growing in the campus of CAU. From 11–12 June 2019 to 17–18 July 2019, eight measurement campaigns of gas-exchange were conducted on the leaflet that we planned to harvest. Gas-exchange was determined with a portable photosynthesis system (LI-6400; LI-COR, Inc., Lincoln, NE, USA) between 9:30 am and 11:30 am. The method of gas-exchange measurements followed Wang et al. (2008) [52]. In detail, before the measurement of gas-exchange, it takes about 5 s to stabilize after the leaf was inserted in the cuvette. Then the measurements were carried out under the conditions of a standard 450 mmol mol^−1^ CO_2_ concentration at a flow rate of 500 mmol s^−1^ above saturation in photo flux density of 1600 mmol m^−2^ s^−1^. The temperature of the leaf varied from 29.5 to 30.5 °C during the entire period of gas-exchange measurements.

### 4.3. Measurements of Leaf Morphology, N Concentrations and Carbon Isotope Ratios 

Carbon isotope ratios (δ^13^C) and N concentration were measured for all leaves collected in this study; but only those plant leaves collected from the second and third sampling campaigns were determined for leaf morphology. For measurement of leaf morphology, the leaves were returned to the laboratory as quickly as possible after sampling, kept at 4 °C. Measurements of Leaf area and thickness were completed within 4 h after samples collection. Leaf area was measured by a scanner (microtek Phantom v700plus). The methods of leaf δ^13^C and leaf N followed Li et al. (2016) [10]. Leaf N was measured using an elemental analyzer (Flash EA1112, CE Instruments, Wigan, UK), with a combustion temperature of 1020 °C. Urea, obtained from the International Atomic Energy Agency (IAEA), was taken as the laboratory standard matter. The standard deviation for this N measurement was less than 0.1%. Leaf δ^13^C was determined on a Delta Plus XP mass spectrometer (Thermo Electron GmbH, Bremen, Germany) coupled with an elemental analyzer in continuous flow mode. About 250 μg of ground leaf material was included in a tin capsule, which was placed in the elemental analyzer, where the sample was combusted at a temperature of 1020 °C. Glucose, obtained from the IAEA, was used as the laboratory standard matter. The standard deviation of δ^13^C was 0.15‰. δ^13^C was reported in the standard notation relative to the Vienna Pee Dee Belemnite standard.

Carbon-isotope discrimination (Δ) of plants was obtained by the following formula (Farquhar et al., 1982) [2]:

(1) $$\Delta =\frac{\delta^{13}C_{\mathrm{air}}{-\delta }^{13}C_{\mathrm{plant}}}{{1+\delta }^{13}C_{\mathrm{plant}}/1000}$$

where δ^13^C_plant_ is the measured δ^13^C value of leaf, δ^13^C_air_ is the δ^13^C value of ambient CO_2_. The δ^13^C_air_ was assumed to be −8.65‰, −8.68‰ and −8.77‰ for 2017, 2018 and 2019, respectively (http://www.esrl.noaa.gov/gmd/ccgg/globalview/co2c13_intro.html, accessed on 3 February 2020).

### 4.4. Quantification of Leaf Protein

Leaf-protein concentration of only the leaves obtained from the third sampling was quantified by Shanghai Jining bioscience and biotechnology company using BCA kit. Protein quantification was not performed on *H. dulcis* because its leaf mass was often small and not enough for this quantification. In addition, for the small leaves of the other five species, we did not conduct protein measurements.

### 4.5. Statistical Analysis

Statistical analyses were conducted using SPSS software (SPSS for Windows, Version 20.0, Chicago, IL, USA). Pearson analysis was used to determine the correlation among N_mass_, N_area_, gas exchange and Δ at a significance level of *p* < 0.05.

## 5. Conclusions

This study explored the relationship between leaf N and carbon isotopic discrimination (Δ), from the three levels of community, population, and plant individual. At the community level, a positive correlation, and a no correlation between leaf N and Δ were found across the study sites. At the population level and ata the individual level, there were positive, negative, and no relationships between leaf N and Δ. The observations strongly suggest that there is no common pattern in the leaf N–Δ relationship and this relationship may be dependent on environmental conditions, species and plant individuals. Furthermore, this observation indicates that long-term leaf-level water-use efficiency (WUE) may not often be associated with leaf N in the metabolic process of carbon, N, and water in leaves. Compared with previous studies, this study effectively revealed the relationship between leaf N and Δ because the influences of changing environments, inter-species difference, and inter-individual difference on the relationship were minimized. One of the mechanisms accounting for the lack of a relationship was that most plants did not invest large amounts of nitrogen into photosynthesis. In addition, the present study also observed that for most species, there was no correlation between leaf N and photosynthetic rate; A, and the variations in A were mainly driven by stomatal conductance, g_s_.

## Data Availability

The data that support the findings of this study are available from the corresponding author upon reasonable request.

## Appendix A

**Table A1:** Correlations between leaf Nmass and leaf protein concentration for five plants derived from the third sampling campaign.

Species	Number of Samples	*p*	r
*S. oblate*	16	0.812 ^ns^	0.065
*A. chinensis*	14	0.847 ^ns^	0.057
*V. sargentii*	16	1.000 ^ns^	0.000
*V. rhytidophyllum*	14	0.048 *	0.536
*C. trichotomum*	10	0.845 ^ns^	0.071

Note: *H. dulcis* had small leaf size, so its protein was not measured; in addition, protein-measurements were not also conducted on those leaves with small size of the other five plants. * indicates a significant correlation (*p* < 0.05), ^ns^ indicates no correlation.
